# Molecular epidemiology of camel trypanosomiasis based on ITS1 rDNA and RoTat 1.2 VSG gene in the Sudan

**DOI:** 10.1186/1756-3305-4-31

**Published:** 2011-03-04

**Authors:** Bashir Salim, Mohammed A Bakheit, Joseph Kamau, Ichiro Nakamura, Chihiro Sugimoto

**Affiliations:** 1 Department of Collaboration and Education, Research Center for Zoonosis Control, Hokkaido University, Sapporo 001-0020, Japan; 2 Department of Parasitology, Faculty of Veterinary Medicine, University of Khartoum, 13314 Khartoum-North, Sudan

## Abstract

**Background:**

Internal transcribed spacer one (ITS1) of the ribosomal DNA is known to be a suitable target for PCR-based detection of trypanosomes. The analysis of this region provides a multi-species-specific diagnosis by a single PCR. Using ITS1 primer-based PCR, a cross sectional study was carried out in the period from September to November 2009 on samples collected from 687 camels from geographically distinct zones in the Sudan to detect all possible African trypanosomes, which can infect camels.

**Results:**

The results showed that all PCR-positive camels were infected with a single parasite species; *Trypanosoma evansi*. The highest prevalence, 57.1% (117/205), was observed in the Butana plains of mid-Eastern Sudan and the lowest, 6.0% (4/67), was in the Umshadeeda eastern part of White Nile State. In another experiment, the RoTat 1.2 gene encoding the variable surface glycoprotein (VSG) of *T. evansi *was analyzed for its presence or absence by a polymerase chain reaction (PCR) using *T. evansi *species-specific primers. The study showed that the RoTat 1.2 VSG gene was absent in thirteen out of thirty *T. evansi*-positive samples.

**Conclusions:**

It is concluded that camel trypanosomiasis in Sudan is apparently caused by a single parasite species *T. evansi *and there were no other typanosomes species detected. In addition, the disease is highly prevalent in the country, which strengthens the need to change control policies and institute measures that help prevent the spread of the parasite. To our knowledge, this is the first molecular diagnosis report, which gives a picture of camel trypanosomiasis covering large geographical areas in Sudan.

## Findings

*Trypanosoma evansi *is the most widely distributed pathogenic animal trypanosome, affecting domesticated livestock in Asia, Africa, Central and South America, Europe, and recently a case of human infection has been reported in India making it a potential human pathogen [1].

In arid-semiarid areas in Africa (Somalia, Kenya, Ethiopia, Sudan, Chad, Nigeria and French West Africa), camels are affected most by the parasite. In Central and South America, horses are the main hosts followed by cattle [2].

*Trypanosoma simiae *was identified as the cause of an outbreak in dromedaries in a Kenyan national park, confirming the susceptibility of camels to this pathogen [3]. *T. simiae *was also documented as a camel pathogen in Somalia [4]. Wernery and Kaaden [5] experimentally confirmed that dromedaries were sensitive to *T. brucei *and particularly to *Trypanosoma congolense*.

Many molecular markers are used to detect, differentiate and study trypanosome species. The rRNA internal transcribed spacer one (ITS1) and internal transcribed spacer two (ITS2), which are separated by the 5.8S gene and flanked by the small subunit and large subunit rRNA genes in most eukaryotes are subject to higher evolutionary rates leading to greater variability in both nucleotide sequence and length [6]. The ITS1 and ITS2 spacers have been found to be reliably valuable in more discrete phylogenetic separation of closely related species including piroplasms and their subspecies [7-9] and trypanosomes. Generally, the ITS region has been used extensively in characterization of *T. evansi *[10-13]. The ITS1 region has been successfully used as target for PCR-based detection of trypanosomes by [14,15]. These authors documented specific PCR product length corresponding to each *Trypanosoma *species, which was the base of differentiation among *Trypanosoma *species. For example, *T. congolense *savannah, an ITS1 PCR product is 700 bp, 400 bp for *T. simiae *and 250 bp for *T. vivax*. The product for *T. evansi *and *T. brucei *subspecies was the same size, 480 bp. The variable surface glycoprotein of trypanosomes RoTat 1.2 VSG is a predominant variant antigen type thought to be expressed in all *T. evansi *stocks examined so far [16]. It has been used in routine diagnostic antigen tests such as card agglutination test CATT/*T. evansi*, but negative CATT/*T. evansi *reactions in *T. evansi *parasitaemic animals have been reported [17,18]. Thereafter, it has been shown that a number of *T. evansi *in Kenya were not detected by tests based on RoTat 1.2 VSG gene and four of the isolates lacking the RoTat 1.2 VSG gene [19].

Camel trypanosomiasis is of great concern to Sudan, which possesses the second largest camel population in the world being estimated at nearly 4,623,000 heads (Annual Report of Ministry of Animal Resources and Fisheries, Sudan, 2010). Although the disease is generally believed to be caused by *T. evansi*, other species cannot be ruled out pertaining to the wide distribution of hosts and the various trypanosome species potentially present in hosts, or vectors. Previous immunological studies which were carried out in Sudan [20,21] would not be able to survey camels for other trypanosome species, and this an advantage of the ITS1-PCR method used here.

We undertook this study to provide information on the disease prevalence, its local enzootic situation and the possible causative agents in camels from geographically distinct regions.

### Sample collection

**Figure 1:**
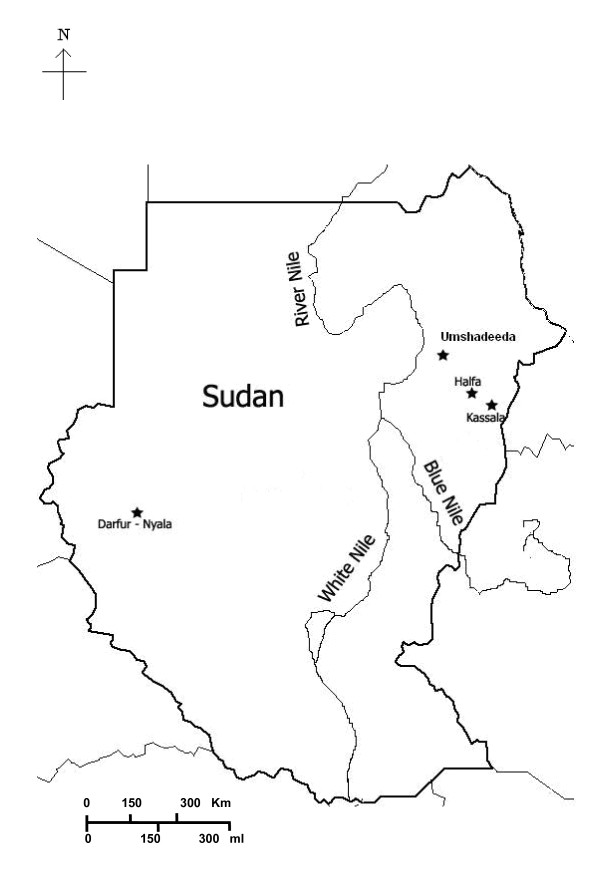
**Map of Sudan. Localization of sampling areas are shown in black stars**.

Blood spots on Whatman FTA^® ^Card (Whatman, Inc.) were collected in a surveillance conducted in autumn 2009 (September 20 to November 10). Camels were sampled from four geographically distinct areas in Sudan. The samples numbers and coordinates of their collection locations are shown in Table 1 & Figure 1.

**Table 1:** The prevalence of *T. evansi *from camel samples and their location coordinates using ITS1-PCR detection in the Sudan

Location	Number of samples	Latitude/Longitude	Prevalence	**95% CI**^**+ **^**for prevalence**	
				Lower	Upper
Kassala	50	15°30'N 36°00'E	24.0% (12/50)	13.1	38.2
Halfa "Butana region"	205	15°19'N 35°36'E	57.1% (117/205)	50.0	64.0
Umshadeeda	67	17°01'N 34°94'E	6.0% (4/67)	1.6	14.6
¤South Darfur	365	12°02'N 24°58'E	7.1% (26/365)¢	4.7	10.3
			35.6% (130/365)*	30.7	40.8

**Total**	**687**				

¤The actual number of camels screened was 365, each five camels' blood were pooled in on spot onto FTA-card

¢Assuming one camel infected (minimum prevalence)

*Assuming all camels infected (maximum prevalence)

+ = confidence interval

### Microscopic examination and DNA extraction

A total of 687 blood samples were collected from camels in FTA cards (Whatman FTA^® ^Classic Cards, Whatman, UK). Of these, 365 samples from Darfur were collected in a way that each five were pooled onto one spot of the FTA-card, which reduced the number of spots to 73 samples (Table 1). DNA was extracted from five punches to ensure correct estimation of prevalence as shown by [22]. DNA was eluted from the FTA cards using a modified methanol fixation method as described by [23]. Extracted DNA samples were stored at -20°C until used.

### PCR

Extracted genomic DNA of 687 samples were subjected to a PCR test, which amplifies the ITS1 region of the rDNA gene of all African trypanosomes by using ITS1 CF/BR [14] FP: 5'-CCGGAAGTTCACCGATATTG-3', RP: 5'-TGCTGC GTTCTTCAACGAA-3'. The 480 bp PCR product was amplified using GoTaq^® ^Colorless Master Mix, 2X (Promega Co. USA) in a 10 μl total volume. Each reaction included 5 μl GoTaq^® ^Colorless Master Mix, 10 mM of each primer, 1 μl RNase-free water and 2 μl extracted DNA. Thermocycling profile started with initial hold for 2 min at 95°C, followed by 35 cycles of 95°C for 30 sec, 58°C for 30 sec. and 72°C for 1 min and final extension step of 5 min. at 72°C. PCR products were electrophoresed in 2% agarose Zebra (BioTools Inc, Japan) in (Tris-acetate EDTA) buffer and stained using GelRed dye (Biotium, Inc. Hayward, CA Biotium, Inc., USA) before being visualized under UV light.

Thirty positive ITS1 *T. evansi *isolates were further subjected to PCR test specific for *T. evansi*, in which a primer set that amplifies 151 bp of the *T. evansi *RoTat 1.2 VSG gene fragment was used [24]. This was TeRoTat920F 5'-CTGAAG AGGTTGGAAATGGAGAAG-3' and TeRoTat1070R, 5'-GTTTCGGTGGTTCTGTTGTTG TTA-3'. The reaction conditions and thermocycling profile were as mentioned above.

The differences in prevalences between different regions were computed using the Chi-square test with SigmaXL Version 6 (SigmaXL Inc., USA). All statistics were considered significant at *P ≤ 0.05*.

**Figure 2:**
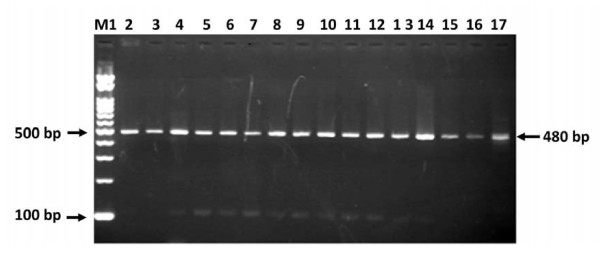
**Zebra agarose gel electrophoresis of field *T. evansi *DNA samples (50 ng) amplified with ITS1 CF and BR**. Lane M1 100 bp marker, lane 2 to 17 *T. evansi *from different locations.

The ITS1-PCR performed to screen camels from four regions in the Sudan (Figure 2), showed higher prevalence in Halfa "Butana region" of 57.1% (117/205) with lower prevalence of 6.0% (4/67) recorded in Umshadeeda (Table 1). Samples from Kassala showed prevalence of 24.0% (12/50) and those from South Darfur showed prevalence of 35.6% (26/73) taken into account that the actual number samples from Darfur was 356 samples (Table 1). Samples were first tested using agarose S electrophoresis for visualizing positive samples. The presence or absence of fragment size differences in the positive *T. evansi *samples was then tested by using 2% agarose Zebra (BioTools Inc, Japan) with high resolution capability of differentiating small base pairs differences. No size difference was observed in all samples (Figure 2).

**Figure 3:**
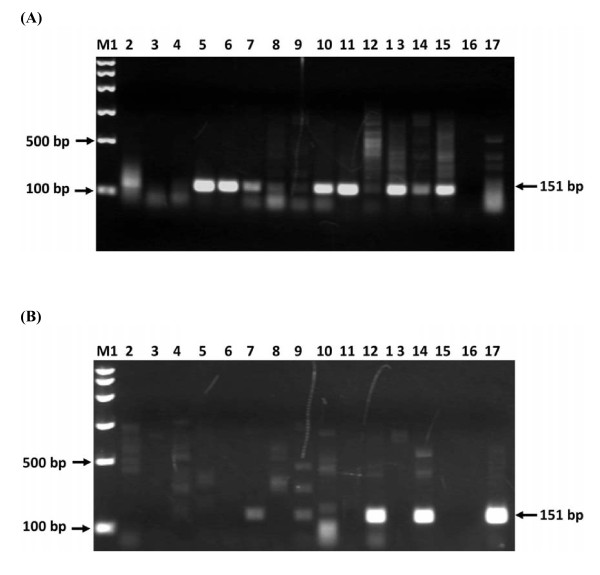
**Agarose gel electrophoresis of field *T. evansi *DNA samples (50 ng) amplified with species specific primers TeRoTat920Fand TeRoTat1070R for *T. evansi *based on RoTat 1.2 VSG gene**. **A**: Lane M1: 6 band marker. All other lanes are RoTat 1.2 VSG gene positive (8, 9, 12 and 17 weak positives; 8 & 9 from South Darfur); except lane 3, 4 and 16 were shown RoTate 1.2 VSG negative from Khowai. Samples shown here are from geographically different areas. **B**: Lane M1 6 band marker. Lanes 2-15 have camel samples. 7 and 9 are weak positives, 12 and 14 are strong positives, rest are negative, ie. 2-6, 8, 10, 11, 13 and 15. Lane 16 and 17 are control negative and positive respectively. (All samples were from Darfur). Extra bands in both gels are non-specific bands.

PCR amplification was carried out targeting RoTat 1.2 VSG gene on 30 ITS1-positive *T. evansi *indicated that 13 out of these were negative for RoTat1.2 (Figure 3A and 3B). Figure 3A showed 16 samples from different geographical regions, of which 3 samples were shown RoTate 1.2 VSG negative. Figure 3B shown 14 samples all from South Darfur, of which only 4 samples where RoTate 1.2 VSG positive.

There was a significant difference in prevalence between the regions. The prevalence of *T. evansi *was significantly higher in Halfa (Butana region) than in Kassala and Umshadeeda (*p < 0.001 *and *p = 0.006 *respectively), while in Halfa (Butana region) the prevalence was significantly higher than in Umshadeeda (*p < 0.001*). The prevalence of *T. evansi *in South Darfur (when assuming 1 in 5 camels infected) was significantly different from Kassala and Halfa (Butana region) (*p < 0.001 *for both) but was not significantly different from Umshadeeda (*p = 0.733*). In contrast, when assuming that all the five camel pooled blood samples were infected, the prevalence was found be significantly different from Halfa (Butana region) and Umshadeeda region (*p < 0.001 *for for both), but not significant from Kassala (*p = 0.104*).

The ITS1-PCR detection method constitutes a powerful molecular diagnostic tool for *T. evansi *detection and discrimination from other trypanosomes in one PCR that is currently available for molecular epidemiological studies of pathogenic African trypanosomes.

Although camels are largely kept without close association with other animals and do not often share the same husbandry conditions as cattle, nonetheless, they do come into contact with donkeys, goats, sheep and horses in the Sudan. The existence of carrier animals in the vicinity of susceptible camels makes transmission by biting flies possible. Therefore, we hypothesized that we might find additional species of trypanosmes apart from *T. evansi *when using ITS1 CF and BR [14]. The ITS1 results clearly revealed that all camels in the investigated areas were infected with trypanosomes of the subgenus *Trypanozoon*, most likely *T. evansi*. Indeed, most samples were also positive with RoTat 1.2 PCR, which is specific for *T. evansi*. However, the possibility of finding tsetse transmitted trypanosomes in naturally infected camels still cannot be excluded especially when animals are raised within, or came into vicinity of other hosts within the tsetse fly zone. This will need further investigations.

The higher prevalence rate which was detected in Butana plains of mid-Eastern Sudan is in agreement with the findings of Elamin et al. [21], who showed the higher prevalence was in the dry season (November to May) in the area. They also found that the parasitaemia was higher in the dry season than in the wet season (June to October) possibly due to the increased activity of biting flies. Our study, which was carried during a similar period (dry season, late September to November), supports the idea of congregation of camel herds at the scarce water holes, which brings the animals into close proximity and facilitates efficient transmission of the parasite by the flies. Other factors which presumably predispose to high infection and probably patent parasitaemia are prolonged drought and a low plane of nutrition during the later part of the dry season. It is worth to mention that the dry season of 2009 was severe in term of pasture availability. Conversely, lower prevalence rates in camels from the Umshadeeda region, eastern part of White Nile State, could be linked to the low fly densities in these areas [25].

These data show that wet film studies detected a much lower prevalences than the ITS1-PCR studies reported here. This raises the question as to whether these differences represent differences in sampling sites or, more importantly, whether the wet film technique is underestimating the prevalence of *T. evansi *infection in camels in Sudan. This question still remains to be addressed. In the case of Darfur, although the prevalence was not accurate because it was estimated per 5 samples, the current results could give an overview of the disease status in the region.

The absence of RoTat 1.2 VSG gene from some *T. evansi *in Sudan has an implication that direct card agglutination test CATT/*T. evansi *based on the predominant variable antigen-type (pVAT) RoTat 1.2 VSG gene has some drawbacks because a considerable number of positive cases will be missed. Most of RoTat 1.2 VSG gene negative samples found during this study were from Darfur region, but also other regions have shown *T. evansi *RoTat 1.2 VSG gene negative results. Further investigations are required to examine the suitability of implementing RoTat 1.2 VSG gene PCR and CATT/*T. evansi *in epidemiological surveys or diagnosis of trypanosomiasis in Sudan.

In conclusion, the study showed out that only *T. evansi *was detected in camels in the Sudan and no other trypanosomes have been detected. In addition, some samples tested negative by RoTat-PCR and positive by ITS1, so that ITS1-PCR is recommended.

## Competing interests

The authors declare that they have no competing interests.

## Authors' contributions

BS carried out the molecular genetic analyses, participated in the data analysis, did the field collection, participated in the statistical analysis and drafted the manuscript. MAB was involved in field collection and helped to draft the manuscript. JK participated to draft the manuscript. IN participated to draft the manuscript. CS helped to conceive the study, participated in its design, assisted in obtaining funding and helped to draft the manuscript. All authors read and approved the final manuscript.
